# Epigenetic Peripheral Biomarkers for Early Diagnosis of Alzheimer’s Disease

**DOI:** 10.3390/genes13081308

**Published:** 2022-07-22

**Authors:** Chiara Villa, Andrea Stoccoro

**Affiliations:** 1 School of Medicine and Surgery, University of Milano-Bicocca, 20900 Monza, Italy; 2 Department of Translational Research and of New Surgical and Medical Technologies, Medical School, University of Pisa, 56126 Pisa, Italy; andrea.stoccoro@unipi.it

**Keywords:** epigenetics, Alzheimer’s disease, biomarkers

## Abstract

Alzheimer’s disease (AD) is a progressive neurodegenerative disorder and represents the leading cause of cognitive impairment and dementia in older individuals throughout the world. The main hallmarks of AD include brain atrophy, extracellular deposition of insoluble amyloid-β (Aβ) plaques, and the intracellular aggregation of protein tau in neurofibrillary tangles. These pathological modifications start many years prior to clinical manifestations of disease and the *spectrum* of AD progresses along a *continuum* from preclinical to clinical phases. Therefore, identifying specific biomarkers for detecting AD at early stages greatly improves clinical management. However, stable and non-invasive biomarkers are not currently available for the early detection of the disease. In the search for more reliable biomarkers, epigenetic mechanisms, able to mediate the interaction between the genome and the environment, are emerging as important players in AD pathogenesis. Herein, we discuss altered epigenetic signatures in blood as potential peripheral biomarkers for the early detection of AD in order to help diagnosis and improve therapy.

## 1. Introduction

Alzheimer’s disease (AD) is a progressive neurodegenerative disorder with a complex etiology and represents the most prevalent cause of dementia in the elderly population worldwide [1]. As the life expectancy of humans is increasing, AD prevalence rate is rising rapidly, making this disorder a growing public health issue with a significant economic burden [2]. AD is clinically characterized predominantly by initial memory deficits and cognitive decline which ultimately affect other functional abilities, including speech, behavior, visuospatial orientation, sleep and the motor system, associated with neuropsychological manifestations [3,4]. AD is conceived as a clinical *continuum* that, starting from the preclinical stage, leads to the development of full-blown dementia, passing through the prodromal stage of mild cognitive impairment (MCI) [5]. The major neuropathological hallmarks of AD include the extracellular deposition of senile plaques composed of the amyloid-β (Aβ) peptides and the intracellular formation of neurofibrillary tangles (NFTs) constituted by hyper-phosphorylated twisted filaments of the microtubule-associated protein tau in the hippocampus [6]. During the disease progression, these pathological changes directly or indirectly activate other mechanisms, such as microglia-mediated inflammation, oxidative stress, mitochondrial dysfunction, calcium-mediated excitotoxicity, and vascular damage [7]. These processes result in neuronal injury, synaptic and neurotransmission dysfunctions, thus leading to the onset of clinical dementia in affected subjects [8,9]. Pathogenic mutations in three genes encoding for proteins involved in the maturation and aggregation of Aβ, namely *PSEN1* (presenilin-1), *PSEN2* (presenilin-2) and *APP* (amyloid precursor protein), are causative of familial early-onset forms of AD affecting individuals under the age of 65 years with an autosomal dominant pattern of inheritance [10]. However, the majority of AD cases are late-onset sporadic forms and usually occur in individuals aged over 65, in which ageing represents the strongest non-modifiable risk factor for the disease [11,12]. These forms have a multifactorial etiology, due to the complex interactions between environmental and genetic factors, with *APOE* ε4 as the major genetic risk factor identified until now [11].

Despite AD prevalence and decades of intensive research into the disease pathogenesis, drugs that can prevent or even halt the progression of this disorder are still lacking in clinical practice [13]. Indeed, the majority of the current therapeutic strategies are merely symptomatic and often present several side effects [14]. Nowadays, the diagnosis of AD is based on clinical examination supported by the detection of Aβ, phosphorylated (p-tau), and total tau (t-tau) protein levels in the cerebrospinal fluid (CSF) of patients in combination with advanced neuroimaging techniques such as positron emission tomography (PET) and volumetric magnetic resonance imaging (MRI) [15]. However, these analyses are high invasive for the patients, poorly available in community health facilities, and relatively expensive for the healthcare system [16]. Moreover, as pathological modifications silently accumulate in the brain over years before the onset of evident symptoms, clinicians face difficulties in diagnosing AD prior to the occurrence of irreversible brain damage [17]. Thus, the current challenge is to search for less costly and intrusive biomarkers associated with pathophysiologic mechanisms and can be used at primary care settings in order to improve the accuracy of clinical AD diagnosis at early presymptomatic stages [18]. In this regard, epigenetics has recently emerged as a promising field for finding novel AD biomarkers, as epigenetic mechanisms have been demonstrated to be dysregulated in several human disorders, including AD [19,20]. By mediating the interplay between the genome and the environment, epigenetic mechanisms could explain the role of non-genetic factors in AD, thus leading to greater understanding of the disease etiology with potential implications also for the disease treatment. Interestingly, epigenetic alterations are also detectable in the peripheral blood of patients, providing easy-to-access biomarkers for the disease [21,22,23].

In this review, we aimed to discuss the main advances in epigenetic biomarkers for the early diagnosis of AD, which could greatly improve the diagnostic accuracy, prognostic assessments, and monitoring the potential response to disease-modifying therapies in AD clinical trials. Moreover, the identification of these biomarkers will lead to better understanding the disease etiopathogenesis and potentially provide novel molecular targets for the development of pharmacological and non-pharmacological therapeutic strategies, able to prevent or slow down the disease course.

## 2. Epigenetic Mechanisms

### 2.1. Overview of the Main Epigenetic Mechanisms

**Figure 1:**
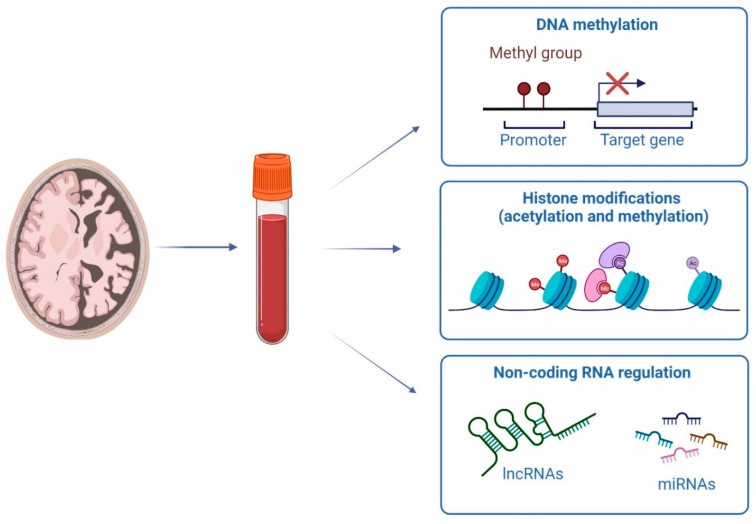
Epigenetic peripheral modifications for the early diagnosis of AD.

The term epigenetics refers to reversible changes able to influence the gene expression through mechanisms that are heritable but without altering the DNA sequence. The main epigenetic mechanisms are DNA methylation, histone modifications, and gene expression regulation mediated by non-coding RNA (ncRNA) [24] (Figure 1).

DNA methylation represents one of the most important epigenetic mechanisms, and has so far been the most studied. It is a dynamic process that takes place during development in multicellular organisms and guarantees the maintenance of normal levels of gene expression. It is involved in numerous cellular processes, including regulation of gene expression, modification of chromatin structure, genomic imprinting, embryogenesis, inactivation of the X chromosome in female mammals and inactivation of transposable genetic elements [25]. DNA methylation is performed by a class of enzymes called DNA methyltransferases (DNMTs), which add a methyl group to a cytosine residue in a CpG dinucleotide context, forming 5-methylcytosine (5-mC). Sites of CpG clusters in the gene promoters are called CpG islands, and when a CpG island is methylated the expression of that gene is usually repressed. By contrast, cytosine methylation in gene bodies could be related to either an active or repressed transcriptional state depending on the tissue in which it occurs [26]. In recent years, it has frequently been observed that the mitochondrial DNA (mtDNA) could also be methylated by DNMTs, and this modification could play a crucial role in the regulation of mtDNA gene expression and of mtDNA replication [27]. Although less frequently and with biological significance not yet clear compared to CpG methylation, DNA methylation can also occur in a non-CpG context, i.e., in CpH sites, where H = A, T, or C, as well as in adenine residues inducing the formation of N6-methyl-2′-deoxyadenosine (6 mA) [28,29]. Characterized from a more functional point of view is the DNA hydroxymethylation of CpG dinucleotides, which is mediated by members of the ten-eleven translocation (TET) protein family, and which is usually associated with increased gene expression. The central nervous system is particularly rich in hydroxymethylcytosine (5-hmC), and this epigenetic mark is likely to be involved in neurodevelopment [30]. A great improvement in our understanding of DNA methylation modifications was derived from the development of several techniques able to detect these modifications.

Histone modifications consist of the post-translational modifications of N-terminal tails of histone proteins, including acetylation, methylation, phosphorylation, ubiquitination and ADP ribosylation. These changes influence the chromatin structure, inducing a heterochromatinic state characterized by condensed chromatin and the repression of gene expression, or an euchromatinic state, characterized by relaxed chromatin which facilitates gene transcription. For example, acetylation neutralizes positive charges of histones, which causes the dissociation of histones from DNA, which has a negative charge, thus facilitating access to the transcriptional machinery, allowing gene transcription [31].

NcRNAs, including microRNA (miRNA, 20–23 nucleotides in length) and long non-coding RNA (lncRNA, length greater than 200 nucleotides) constitute a large and diverse family of non-protein-coding transcripts that modulate gene expression at both transcriptional and post-transcriptional levels [19]. MiRNAs are the most studied ncRNAs, and regulate gene expression in a sequence-specific manner, by binding to the 3′ untranslated region of target mRNA molecules and mediating their post-translational regulation, leading to either degradation or translational inhibition, depending on the degree of sequence complementarity [32]. Mechanisms of action of the lncRNAs are more complex compared to miRNA, as they can interact with mRNA, DNA, protein, and miRNA and consequently regulate gene expression in a variety of ways, including chromatin remodeling, transcriptional activation, transcriptional interference, RNA processing, and mRNA translation [33].

Epigenetic mechanisms finely regulate gene expression levels, and play a fundamental role in embryonic development, differentiation and maintenance of cellular identity, as well as in many other physiological processes. It is now well-recognized that the epigenetic mechanisms are plastic and dynamic processes in response to environmental factors, and that their alteration can contribute to the development of numerous human pathologies [34]. The growing evidence of an involvement of epigenetic modifications in the state of human health and disease has paved the way for the search for epigenetic biomarkers which could be used in clinical practice and for numerous studies aimed at evaluating the contribution of environmental factors in inducing such modifications. In this way, epigenetics is greatly improving patient management, providing biomarkers, of which some are approved by the US Food and Drug Administration (FDA), for diagnosis, prognosis, or response to therapy, as well as for the development of epigenetic-based therapy in several types of cancers [35]. Regarding neurodegenerative diseases, although many potential diagnostic epigenetic biomarkers have been proposed, they have not yet translated into clinical practice. The main limitation is the access to the target tissues, i.e., the central nervous system, meaning that many researchers are focusing their attention on the search for epigenetic biomarkers in tissues that are easier to collect, including peripheral blood. The use of peripheral tissues for the search of epigenetic biomarkers of neurodegenerative diseases could permit the identification of individuals in the preliminary phases of the disorder, and, in longitudinal studies, of individuals who have not yet even developed the disease, thus potentially finding very early biomarkers. In the next sections of the review, the main studies in which epigenetic biomarkers were sought in peripheral tissues of AD patients in the early stages of the disease are reported, particularly in individuals with MCI. The majority of the studies searched for DNA methylation and ncRNA biomarkers, while the research into histone alterations-based biomarkers in the peripheral blood of such type of patients is currently scarce. Indeed, although there is a huge amount of evidence to support the claim that histone modifications are involved in AD pathogenesis, the evidence is derived primarily from studies performed in human post-mortem samples [31]. To the best of our knowledge, until now only one study has investigated histone modifications in the peripheral blood of MCI patients [36]. In that study it was observed that histone acetylation levels were elevated in monocytes of MCI, but not in monocytes derived from AD patients, when compared to the levels observed in control subjects. Interestingly, the authors also observed a significant increase in monocytic histone acetylation in transgenic AD mouse models early during development of the plaque deposition in the brain, further suggesting that this epigenetic modification is an early event during AD pathogenesis [36]. However, further studies are needed to consider peripheral histone acetylation as a candidate biomarker for the early detection of AD patients.

### 2.2. DNA Methylation Investigations in Early AD Stages

DNA methylation studies in tissues derived from patients with AD date back to the early 1990s. Indeed, the first results supporting the involvement of DNA methylation in the pathogenesis of AD were published in 1995, in a study reporting lower methylation levels of the *APP* promoter region in the temporal lobe of an AD patient compared to a non-demented subject [37]. Since then, more than 700 articles have been published on this topic, further supporting the hypothesis that DNA methylation alterations could play an important role in AD pathogenesis. The increase in the number of studies in this field has been due to the development of numerous techniques that have made it possible to analyze DNA methylation in an in-depth and cost-effective manner. A major boost in the study of DNA methylation derived from the discovery that treatment of DNA with sodium bisulfite, which induces deamination of unmethylated cytosines into uracil residues, while 5-methylcytosines are not converted, could be used to easily analyze the state of DNA methylation. Following such treatment, DNA methylation levels can be analyzed by various techniques, which are distinguished mainly in relation to the portions of DNA to be investigated. Investigation of candidate genes/regions are mainly based on two different strategies that are distinguished by the use of primers for methylation-specific PCR reactions, and therefore defined as methylation-specific PCR (MSP), and those that use methylation-independent primers. The latter are the most used and include several techniques, such as the pyrosequencing, considered the gold-standard technique for the study of gene-specific methylation, bisulfite sequencing, and the methylation-sensitive high resolution melting (MS-HRM) technique [38]. Bisulfite-treated DNA could also be used to investigate DNA methylation throughout the genome, by means of whole genome bisulfite sequencing (WGBS), or by means of more cost efficient microarray-based approaches, including Illumina BeadChip microarray that can cover 27,578 (27 K), ~450,000 (450 K), or in its latest generation, ~850,000 (EPIC array) CpG sites [39]. By means of such approaches, differentially methylated positions (DMP) could be identified, namely CpG sites that have different DNA methylation patterns among multiple samples, as well as differentially methylated regions (DMRs), which represent areas of the DNA containing multiple adjacent DMPs. Usually, DMPs and DMRs are further confirmed by using candidate gene approaches.

The first studies that investigated DNA methylation in individuals in the early phases of AD, and in particular in individuals diagnosed with MCI, were published in 2015. In one of these studies, whole-genome DNA methylation was investigated in the peripheral blood of individuals with type-2 diabetes, some of which developed signs of pre-dementia [40]. Authors identified eight CpG sites differentially methylated between converters and non-converters before symptoms at baseline and at 18 months follow-up. One of these probes was located in close proximity to the *RPL13* gene which has been previously associated with AD pathology in post-mortem brains [41,42]. In two other studies, DNA methylation levels were investigated in the peripheral blood of individuals from two Chinese populations, including Uygur individuals, belonging to the Caucasian population, and Han individuals, belonging to Mongolian population [43,44]. In one of these studies, a significant association between *KLOTHO* (a longevity and neuroprotective gene) promoter methylation and MCI in the Han Chinese but not in the Uygur Chinese was observed, and higher *KLOTHO* promoter methylation levels were found in Han MCI patients than Uygur MCI patients [43]. In the other study, no differences in *BDNF* methylation were observed between MCI and control subjects, but the results suggested the existence of different *BDNF* methylation between the two populations, likely due to both genetic background and environmental factors [44]. In the same population, the methylation levels of two genes encoding for opioid receptors, namely *OPRK1* and *OPRM1* [45], were also investigated. No significant associations were observed between the methylation levels of OPRK1 and MCI in both Xinjiang Han and Uygur populations, although the *OPRK1* promoter was significantly hypermethylated in female Han MCI patients [45]. Compared to healthy controls, the methylation levels of one CpG site in *OPRM1* were higher in Xinjiang Uygur MCI, while methylation of the other two CpG sites were lower in Han MCI [45]. In a following study by the same research group including only the Uygur population, it was observed that the methylation levels of *DLST* and *OGG1* genes, involved in citric acid cycle and DNA repair, respectively, were not associated with MCI [46]. However, *DLST* hypomethylation was significantly associated with MCI in the carriers of *APOE* ε4, while among the non-*APOE* ε4 carriers younger than 75, *OGG1* hypermethylation levels were significantly associated with MCI [46]. These studies showed that peripheral blood methylation could be used as a biomarker for MCI, and that it is strongly related to gender, ethnicity, genetic factors, and environmental changes.

In 2016, a study investigating methylation levels of the sortilin-related receptor 1 (*SORL1*) gene, which is involved in the cleavage and trafficking of APP, in the peripheral blood of diabetic patients with MCI, as well as in diabetic patients without MCI and in control subjects, was published [47]. The authors observed that the methylation ratio of MCI patients was significantly higher than that in diabetic patients without MCI and control subjects [47]. In the same year, peripheral blood DNA methylation in the *NCAPH2*/*LMF2* promoter region, two genes involved in mitosis and maturation of lipoprotein lipases, respectively, was found to be significantly decreased in patients with AD and amnestic MCI (aMCI), i.e., MCI with memory impairment, when compared to healthy subjects. These were significantly higher in the AD group compared to MCI individuals [48]. Interestingly, in a following study, *NCAPH2*/*LMF2* methylation levels were found to correlate with hippocampal atrophy [49]. The same authors investigated the promoter methylation levels of *COASY* and *SPINT1* genes, encoding for a carrier of acetyl and acyl groups and for serine protease inhibitors, respectively, which were significantly increased in AD and aMCI compared to control subjects [50]. Particularly, *COASY* promoter region showed to be a high sensitivity and specificity diagnostic biomarker and was associated with dementia severity [50]. The usefulness of *COASY* promoter methylation as an early biomarker of AD was further confirmed in a more recent study by the same authors using a larger sample size [51]. Another study published in 2016 did not detect differences in global DNA methylation levels among AD, MCI and control subjects [52]. On the other hand, methylation levels of *HMOX1* gene, which encodes an enzyme that mediates the degradation of heme, were found to be lower in the peripheral blood of AD patients compared to MCI and control individuals [53]. However, no differences between MCI and controls were observed, suggesting that, although *HMOX1* gene methylation is altered in AD patients, its evaluation is not suitable for identifying individuals in early stages of disease.

In 2017, two studies were published that showed the usefulness of peripheral *BDNF* methylation as an early biomarker of AD. Indeed, increased levels of *BDNF* promoter gene methylation were observed in the peripheral blood of MCI patients compared to control subjects, and were also increased in the MCI patients who converted to AD compared with the non-conversion group at the 5-year follow up point, thus suggesting that peripheral *BDNF* methylation could serve as an epigenetic biomarker for predicting the conversion from MCI to AD [54]. In a following study, the authors observed that the interaction between DNA methylation of a CpG site in the *BDNF* promoter and a SNP in the *BDNF* gene increased the risk of the development of aMCI and its progression to AD [55]. However, the value of *BNDF* methylation as an early biomarker for dementia was questioned by a later study by Fransquet and collaborators, who investigated the association between peripheral blood and buccal *BDNF* gene methylation and incidence of all-cause dementia after a 14-year follow-up [56]. Only weak evidence, that did not survive multiple comparisons, supported the hypothesis that *BDNF* methylation has the potential to be a biomarker for preclinical or diagnosed dementia. The same research group performed a DNA investigation at the genome-wide level in the peripheral blood DNA of 73 individuals prior to dementia diagnosis and 87 cognitively healthy controls, as well as in the peripheral blood of 25 3-year follow-up dementia cases, and 24 controls [57]. The authors found a CpG site differently methylated between dementia cases prior to diagnosis and controls associated with the general transcription factor IIA subunit 1 (*GTF2A1*) gene. When comparing dementia cases vs. controls, no significant differences were detected [57]. In the same cohort, by adopting a candidate gene approach analysis in genes involved in AD, including *APOE*, *APP*, *BDNF*, *PIN1*, *SNCA* and *TOMM40* [21], the authors observed that the average methylation levels of *APOE* and *TOMM40* differed between presymptomatic and control groups, and confirmed no association between *BDNF* methylation and risk of developing dementia [21].

A methylation analysis at the genome-wide level published in 2018 performed on the peripheral blood of 48 subjects, including 24 MCI, found a number of DMPs and DMRs that were associated with cognitive impairment [58]. The most significant DMPs resided in the *BNC1* gene, which encodes a zinc finger protein basonuclin, that has been previously associated with AD [59], while the top DMRs identified resided in genes encoding subunits of the human leukocyte antigen DP receptor, whose altered expression levels have been previously associated with the transition from MCI to AD [60].

Several DMPs and DMRs were also detected in a study published in 2019, performed on the peripheral blood of 45 American-Mexican MCI and 45 control subjects [61]. Particularly, altered methylation levels were found in genes involved in neuronal cell death, metabolic dysfunction, and inflammatory processes. In the same year, an interesting longitudinal study was published considering the impact of both dietary intakes and biomarker statuses of B vitamins that are involved in DNA methylation and oxidative stress on cognitive health, and DNA methylation levels in elderly patients followed for 2.3 years, some of whom developed MCI [62]. The authors observed that inadequate dietary intake of vitamin B12 was significantly associated with accelerated cognitive decline, whereas adequate folate, vitamin B6, and vitamin B12 intakes were significantly associated with better cognitive reserve. The DNA methylation analyses revealed that *NUDT15* and *TXNRD1* were significantly hypermethylated in MCI patients, and significant correlations of hypermethylated sites with serum levels of folate, homocysteine, and oxidative biomarkers were observed, and interactive effects of B vitamins and hypermethylated sites were significantly associated with cognitive performance [62]. By comparing blood whole-genome DNA methylation levels of non-demented individuals who converted to AD dementia and to non-converted elderly individuals, several DMRs have been identified [63]. Interestingly, one of these DMRs included CpG sites close to the transcriptional start site of the *OXT* gene (encoding a precursor protein that is processed to produce oxytocin and neurophysin I) which the authors found to be altered in middle temporal gyrus specimens of AD patients, thus suggesting that altered peripheral blood methylation levels could mirror DNA methylation alterations in the brain tissues of AD patients [63]. Investigation at the genome-wide level in 284 individuals, including 89 nondemented controls, 86 patients with AD, and 109 individuals with MCI, of which 38 progressed to AD within 1 year, identified several CpG sites whose methylation levels were associated with MCI to AD conversion [23].

The studies cited so far recruited individuals characterized only by neurological examinations. However, to clearly established the MCI disease status additional investigations, including CSF and neuroimaging analyses, should be performed. Investigation of *TOMM40*-*APOE*-*APOC2* locus methylation levels in a study population characterized by CSF biomarkers identified different methylation levels between MCI and AD patients compared to control, and showed that methylation levels associated with CSF Aβ levels [64]. In a later study performed on individuals characterized by neurological and neuroimaging analyses, methylation levels of the IV exon of the *APOE* gene were found to be altered in the peripheral blood of MCI patients when compared to control subjects [65]. By using a well-characterized AD population, the so-called ADNI (the Alzheimer’s Disease Neuroimaging Initiative), which includes individuals who underwent imaging measures (MRI, PET) and analyses of AD biomarkers in blood and CSF, several DMPs were found when comparing methylome among AD, MCI and control subjects [66]. The authors observed that DMPs from each pairwise comparison were associated with genes involved in brain-related pathways. The DMP that had the strongest association with MCI vs. controls was annotated to *CLIP4* (which is a member of the CAP-Gly Domain Containing Linker Protein Family), which was also negatively associated with mini-mental state examination (MMSE) score. The most strongly associated DMP with MCI vs. AD was annotated to *NUCB2* (nucleobindin 2), a calcium ion binding protein that regulates intracellular calcium levels, which also negatively associated with MMSE score. In addition, *BIN1* and *BDNF* were among the significant DMP hits [66]. Using the same study population, two papers identified a gene associated with the conversion from MCI to AD status, the *PM20D1*, which is involved in several processes, including the amide biosynthetic process, cellular amide catabolic process, and the negative regulation of neuron death [22,67]. Of note, from longitudinal data, it was shown that initial promoter hypomethylation of *PM20D1* during MCI and early-stage AD is reversed to promoter hypermethylation in late-stage AD [22]. More recently, another investigation at genome-wide levels performed on 34 cognitively healthy individuals of which 17 developed dementia after 4 years, identified several methylated regions that associate with conversion to dementia, including loci associated with *PM20D1* [68].

Using a population characterized by neurological examination and CSF biomarkers, one study focused on subjects with subjective cognitive decline (SCD), an earlier stage of AD compared to MCI, which were characterized by lower *BIN1* methylation levels when compared with cognitively normal individuals [69]. Furthermore, *BIN1* methylation correlated with CSF biomarkers, particularly in the SCD group. The *BIN1* gene, encoding for the bridging integrator 1, is the second most important susceptibility gene for late-onset AD after the *APOE* gene, and interestingly, two large independent autopsy studies showed that there were methylation changes in the *BIN1* of the AD patient’s brain, accompanied by high expression of *BIN1* [41,70]. We recently identified mtDNA higher D-loop methylation levels, which regulates both mtDNA replication and gene expression, in MCI patients characterized by neurological examination, CSF biomarkers, and neuroimaging analyses compared to control subjects and AD patients at both early and advanced stages of the disease [71]. Moreover, higher D-loop methylation levels were detected in controls compared to AD patients in advanced stages of the disease, but not in those at early stages. Interestingly, D-loop methylation levels negatively correlated with CSF concentrations of p-tau.

These studies clearly suggest that peripheral DNA methylation could be sensitive to AD pathogenesis progression, and could provide peripheral biomarkers of disease. Methylation of several genes have been proposed as potential early biomarkers of AD, including *RPL13*, *KLOTHO*, *SORL1*, *NCAPH2*/*LMF2*, *BDNF*, *OXT*, *COASY*, *APOE*, *BIN1* and *PM20D1* (Table 1). However, it is still difficult to propose a peripheral DNA methylation biomarker with the data obtained so far, as further confirmatory experiments are needed. Among the most investigated genes is the *BDNF*, in which methylation levels have been found to increase in MCI patients by a research group [54,55], but no significant alteration were detected by others [21,44,56]. Therefore, further analyses are needed to better characterize the potential usefulness of *BDNF* methylation as an early biomarker of AD. Moreover, methylation levels of the *APOE* gene have been frequently investigated in the peripheral blood of patients in the early stages of AD, and all the studies performed so far identified differential methylation between MCI or presymptomatic dementia patients and the control group, suggesting its usefulness as an early biomarker for AD [21,64,65]. The *PM20D1* gene deserves a special mention, as its methylation levels have been found to be altered in the peripheral blood of MCI patients by three different research groups [22,67,68]. Interestingly, previous investigations showed strong associations between *PM20D1* gene methylation and AD. Sanchez-Mut et al., by comparing DNA methylome data obtained in different studies performed on brain samples, observed that the *PM20D1* gene displayed promoter hypermethylation in patients with advanced-stage AD when compared to healthy controls [72]. They also found that PM20D1 is a methylation and expression quantitative trait locus (QTL) coupled to an AD-risk associated haplotype (including SNPs rs708727 associated with the *SLC41A1* gene and rs960603 associated with the *PM20D1* gene). Furthermore, *PM20D1* was increased following AD-related neurotoxic insults at symptomatic stages in the APP/PS1 mouse model of AD and in human patients with AD who are carriers of the non-risk haplotype. In line with this, genetically increasing or decreasing the expression of PM20D1 reduced and aggravated AD-related pathologies, respectively, thus suggesting that in a particular genetic background, PM20D1 contributes to neuroprotection against AD [72]. In a following study, the authors further confirmed that frontal cortex *PM20D1* DNA methylation and expression are significantly correlated with the AD pathology [73]. More recently, an investigation performed on the blood DNA of 32 nonagenarians individuals, including 21 cognitively healthy subjects and 11 AD patients, found that *PM20D1* methylation was increased in AD individuals, and that methylation levels were associated with rs708727, but not with rs960603 [74]. These studies clearly highlight that the methylation status of *PM20D1* is altered in AD, and that the methylation status is also dependent on the genetic background of the individuals. More interestingly, *PM20D1* methylation status seems to be highly sensitive to disease progression and thus is a promising peripheral biomarker for early detection of AD.

### 2.3. Non-Coding RNAs

Accumulated evidence has demonstrated that some ncRNAs play important regulatory roles in the key signaling pathways associated with AD pathology, including Aβ aggregation/production, tau hyperphosphorylation, neuroinflammation, synaptic failure and mitochondrial dysfunction [75,76,77]. The majority of studies investigated dysregulated miRNAs as peripheral biomarkers for early AD, but also lncRNAs are emerging as possible epigenetic players able to detect the disorder in early stages [19]. Different approaches have been developed to study ncRNA expression. The most commonly used method to detect the expression of specific ncRNAs is real-time PCR. By means of microarray analysis, which involves the use of nucleotide probes complementary to the series of miRNAs of interest, it is possible to analyze a large number of miRNAs and their regulation in a single experiment. Next generation sequencing platforms are also available for sequencing RNA molecules, thanks to which it is possible to discover the deregulation of new miRNAs [72].

#### 2.3.1. MicroRNAs

Given their high specificity, repeatability, accuracy and stability, several studies have been performed to detect dysregulated miRNAs in blood capable of discriminating early disease onset, especially MCI condition, from fully developed AD and/or healthy individuals [78,79]. Concerning MCI, an interesting study demonstrated that serum miRNAs (hsa-let-7g-5p, hsa-miR-107, and hsa-miR-186-3p), together with diet and gut microbiota composition, act as combinatorial biomarkers to successfully distinguish MCI subjects from controls [80]. A miRNA profiling study performed with Solexa sequencing assay and the subsequent validation by quantitative reverse transcription real-time PCR (RT-qPCR) identified markedly reduced levels of miR-31, miR-93, miR-143, and miR-146a in the serum of AD patients. Interestingly, significantly decreased concentrations of miR-143 combined with high levels of miR-93 and miR-146a were found in MCI subjects compared with healthy controls [81]. Moreover, other authors reported that two sets of plasma miRNAs, namely the miR-132 (miR-128/miR-491-5p, miR-132/miR-491-5p and miR-874/miR-491-5p) and miR-134 families (miR-134/miR-370, miR-323-3p/miR-370 and miR-382/miR-370) are able to successfully differentiate MCI from age-matched controls with high specificity and sensitivity. Importantly, the identified biomarker pairs could also detect MCI at the asymptomatic stage before the clinical diagnosis and age-related brain changes [82,83]. Additionally, two sets of miRNAs in plasma, consisting of hsa-miR-191 and hsa-miR-101, and hsa-miR-103 and hsa-miR-222, have been shown to have great accuracy for MCI detection, attaining the highest area under the curve (AUC) value of 0.962 [84]. Furthermore, in a panel of 15 differentially expressed miRNAs selected in the pilot screening according to the protein putative targets involved in AD, six plasma miRNAs showed the highest fold changes as well as specificities and sensitivities to detect AD at the early stage from healthy controls [85].

In regard to aMCI, some studies have been performed. Circulating miR-34c in serum was found to be significantly increased in patients with aMCI compared with age-matched controls, showing a 64.62% sensitivity and 100.0% specificity by ROC curve analysis. Interestingly, a positive correlation between relative expression levels of miR-34c and MMSE scores has also been observed, further suggesting that it may be a predictive biomarker for aMCI diagnosis in a clinical setting [86]. Similarly, another study revealed the extensive capability of plasma miR-107 to differentiate aMCI patients from healthy controls with sensitivity of 98.3% and specificity of 82.7% [87]. Furthermore, among four aberrant expressed miRNA detected in plasma samples of AD, miR-43a-5p and miR-545-3p were also able to discriminate preclinical AD from AD and control subjects, although these results lacked of a validation cohort [88]. Finally, a microarray sequencing performed on different discovery, analysis and validation cohorts provided a signature consisting of five plasma miRNAs, including miR-1185-2-3p, miR-1909-3p, miR-22-5p, miR-134-3p, and miR-107, able to discriminate aMCI from controls with outstanding accuracy [89].

Collectively, these data reported promising results in the use of peripheral miRNAs as potential biomarkers for AD diagnosis at early stages. However, clearly distinguishing between MCI and AD still remains a challenge. Although plasma levels of miR-92a-3p, miR-181c-5p and miR-210-3p were found to be more elevated in MCI than AD, they both showed a significant upregulation in comparison to healthy controls [90]. In a similar way, levels of miR-483-5p were higher in the plasma of MCI and AD than controls, but they were low in AD patients when compared to MCI subjects, thus making difficult the discrimination between the two stages [91].

#### 2.3.2. Long Non-Coding RNAs

The widely investigated lncRNA in AD is BACE1-AS, which is transcribed by RNA polymerase II from the antisense strand of β-secretase 1 (*BACE1*) gene, encoding the essential enzyme involved in the processing of APP into neurotoxic Aβ peptides. BACE1-AS can pair to *BACE1* mRNA, inducing modifications in its secondary or tertiary structures [92]. This binding results to an increase in mRNA stability and translation, promoting thus additional Aβ generation [93]. High levels of lncRNA BACE1-AS in plasma were found to be higher in AD patients than healthy controls, suggesting its role as potential biomarker for AD diagnosis [94]. Subdividing the patient group into pre-AD and full-AD according to the disease progression evaluated by MMSE, another study found that plasma levels of BACE1-AS were low in the pre-AD subgroup compared with full-AD subjects and healthy controls. Additionally, ROC curve analyses revealed that BACE1-AS can discriminate between all these groups with high specificity and sensitivity, strengthening its potency as a predictive biomarker [95]. These data are in line with results coming from in vivo studies: young-aged mice, mimicking the early stages of AD, displayed low levels of *BACE1* mRNA and BACE1-AS where aged mice exhibited an increased expression of these transcripts. It can be speculated that the hippocampus is responsible of neuroplastic response during the initial phases of AD through the modifications in own gene expressions [96]. However, the progressive diminution of neural plasticity during aging makes these compensatory mechanisms ineffective against AD, leading to an increase in *BACE1* and BACE1-AS expression [93,97].

Another lncRNA proposed as a possible peripheral biomarker for the early detection of AD is 51A. It is transcribed by RNA polymerase III from the antisense strand mapped onto the first intron of the *SORL1* gene [98]. The lncRNA 51A acts as a regulator of *SORL1* alternative splicing by promoting the shift from the expression of the canonical long variant A towards the alternatively spliced isoform. This results in impaired APP processing with a consequent increase in Aβ deposition [99]. Plasma levels of 51A were found to be up-regulated in sporadic AD patients compared with controls and negatively correlated with MMSE scores, suggesting its potential use as a stable biomarker for AD diagnosis [100].

A very recent study performed a lncRNA expression profile in plasma samples isolated from AD individuals at different stages, including preclinical-AD, MCI and advanced-AD compared with matched healthy controls [101]. Among 90 screened lncRNAs, the authors found significantly higher levels of nuclear-enriched abundant transcript 1 (NEAT1) and brain cytoplasmic (BC200) in AD subjects than the control group with sensitivity of 72% and 60%, and specificity of 84% and 91%, respectively, evaluated by ROC curve analysis. Interestingly, the study revealed that plasma levels of NEAT1 are able to distinguish MCI and advanced-AD from healthy controls, indicating that this lncRNA may represent a biomarker for AD diagnosis, as previously observed in the brain tissue of AD post-mortem patients [102] and in animal models of AD [103]. NEAT1, transcribed by RNA polymerase III from multiple endocrine neoplasia locus (MEN1), is aberrantly expressed, mainly upregulated, in non-cancerous pathological conditions, promoting the development and progression of AD [104]. An upregulation of NEAT1 prompted the ubiquitination and degradation of PTEN-induced putative kinase 1 (PINK1), leading to the inhibition of autophagy signaling that resulted in increased Aβ accumulation and cognition dysfunction in an APP/PS1 mouse model [103]. In addition to NEAT1, the lncRNA profiling study identified the aberrant expression of BC200 [101]. It is transcribed by RNA polymerase III in the cell body of neurons and then transported to the dendrites during synaptogenesis where it acts as a translational regulator in the modulation of long-term synaptic plasticity [105]. Interestingly, the authors found that BC200 levels are significantly increased in the plasma of preclinical-AD subjects compared with the control group, suggesting it as promising biomarker in the early detection of the disease [101]. These findings are in agreement with previous research suggesting upregulated BC200 levels in the early stage of AD [106,107].

Although these data are promising (Table 2), more research is still needed for the routine clinical use of lncRNA as a peripheral blood biomarker for early diagnosis of AD. Combining lncRNA levels with other circulating biomarkers and morphological parameters of the brain could improve the accuracy of the disease diagnosis, as already reported [108].

## 3. Limitations and Challenges

**Figure 2:**
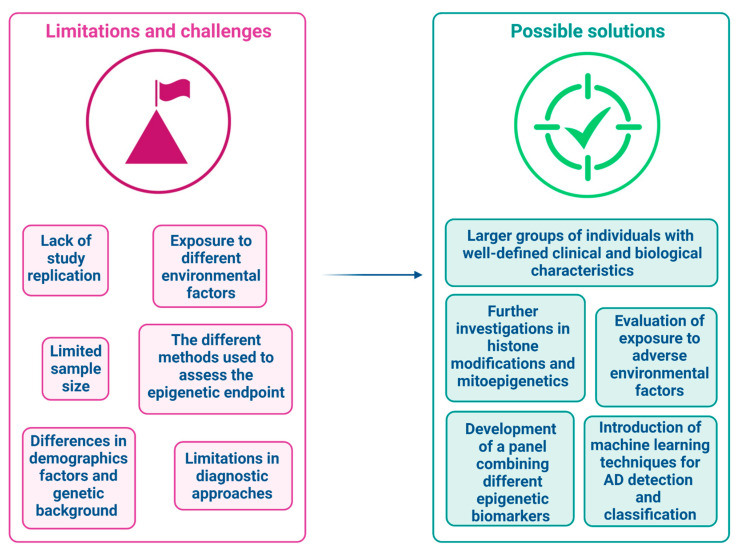
Use of epigenetic peripheral biomarkers for early diagnosis of AD: main limitations, challenges and possible solutions.

Although, investigations with individuals in the prodromal stages of the disease are still scarce, results obtained so far suggest that epigenetic alterations may be an early event in AD etiology and could be detected even in the early stages of the disease in peripheral tissues (Table 1 and Table 2). Indeed, we reported several differentially methylated loci and differentially expressed ncRNAs detected in the peripheral tissues of patients in early stages of AD that can potentially be used as early biomarkers. Nevertheless, it should be outlined that several limitations and challenges in those studies did not yet allow the identification of a valid epigenetic biomarker for early diagnosis of AD (Figure 2). Indeed, the majority of the findings are related to studies that have not been replicated by independent research groups, and need to be confirmed. Moreover, there are discrepancies in some results, as reported, for example, with regards to *BDNF* methylation, which has been proposed as a candidate peripheral biomarker by a research group [54,55], but not by others [21,44,56]. Several factors may contribute to the discrepancies in the findings or in the failure to replicate the results, including the often-limited sample size of the study populations, demographics factors, genetic background, exposure to different environmental factors and, particularly regarding DNA methylation studies, the different methods used to assess the epigenetic endpoint. As the majority of authors focused their studies on a single molecule, the development of a panel combining epigenetic biomarkers from different categories could improve the diagnostic accuracy of early AD. Another important issue to consider concerns the diagnostic approach. Indeed, in the majority of the studies, the diagnosis of MCI was based only on neurological examination and cognitive tests. However, determining the underlying cause of cognitive impairment with the help of CSF and neuroimaging markers is particularly useful in the pre-dementia stage of MCI, as it provides important prognostic information and allows to discrimination between the patients with MCI due to AD and MCI that do not have dementia, including impairments resulting from head trauma, substance abuse, or metabolic disturbance [109], as well as distinguishing individuals with physiologically age-related cognitive decline. Therefore, it is possible that many of the MCI patients enrolled in the study performed are not MCI patients with clinic-pathological characteristics of AD, leading to discrepancies in results, as the methylation alteration detected should be related to different pathogenesis.

Given these limitations, further investigations are needed for the use of epigenetic peripheral biomarkers to detect early AD in the routine clinical application.

## 4. Conclusions and Future Perspectives

Early detection of individuals in the AD *continuum* is of outmost importance as this can lead to improvement in a patient’s management and in the discovery of new therapies that can be administered before the symptoms’ onset. Nowadays, there are several methods used in healthcare that are able to identify patients on the AD *spectrum*, already in early stages of the disease, which mainly rely on the analysis of CSF biomarkers and on imaging techniques. However, the available approaches are expensive, relatively invasive for the patients, and have low sensitivity and specificity, thus limiting their use as screening tests. There is, therefore, a need for supplemental biomarkers that permit the monitoring of AD progression over time and that can reflect the response, if any, to therapeutic interventions. Epigenetic biomarkers, which are greatly sensitive to environmental factors and to genetic background, have been proposed and are currently used as peripheral biomarkers for several human complex diseases, particularly in cancer management [35]. However, several studies have been produced in recent years underlying the pivotal role that epigenetics play in the etiology of AD, demonstrating that many efforts are being made to identify peripheral epigenetic biomarkers for AD as well. Unfortunately, the majority of these studies have been conducted in the brain tissues of deceased AD patients or in the peripheral blood of AD patients in advanced stages of the disease, when the disease is too advanced to be able to intervene.

Results of the studies included in the current review show that several epigenetic marks have been suggested as potential biomarkers for early identification of patients in the AD *spectrum*. Methylation levels of *BDNF*, *APOE* and *PM20D1* seem to be promising peripheral biomarkers able to distinguish individuals in the prodromal stages of the dis-ease. Particularly, methylation of the *PM20D1* gene has been frequently associated with AD pathogenesis, and has been found to be highly sensitive to disease progression. Another promising peripheral biomarker is the methylation of the *BIN1* gene, which has been found altered in SCD patients, indicating that its methylation levels are altered in the very early stages of the disease [69]. Moreover, *BIN1* methylation levels are associated with CSF p-tau and t-tau levels, which are specifically altered in AD pathogenesis and are sensitive to the neurodegenerative process, thus suggesting that this peripheral biomarker could be used to monitor the progression of neurodegeneration [110]. Regarding ncRNAs, there are no promising biomarkers, given the lack of replication studies in independent cohorts.

Although studies included in this review support the potential use of peripheral epigenetic biomarkers to monitor AD pathogenesis in living patients, research in this field is still in its infancy. Future works (Figure 2) should be performed on large groups of well-characterized individuals, with well-defined clinical and biological characteristics, followed over time to observe how progressive cognitive decline correlates with epigenetic biomarkers. In this context, the recent introduction of machine learning techniques for the detection and classification of AD may represent valuable tools for predicting the progression of MCI to early AD [111,112]. Combining MRI, PET and other imaging procedures, together with clinical and neuropsychological assessments, these methods take the disease complexity into account, leading to a more robust classifier of AD [112]. Moreover, as the majority of authors focused their studies on a single molecule, the development of a panel combining epigenetic biomarkers from different categories could improve the diagnostic accuracy of early AD. Until now, only one study focused on the search for histone modifications in the peripheral blood of individuals in the prodromal stages of AD [36], providing encouraging results, and further work could reveal the real usefulness of this epigenetic modification for the early detection of individuals on the AD *spectrum*. Similarly, in recent years we have seen increasing evidence that epigenetic modifications of mitochondrial DNA (mitoepigenetics) also likely play a significant role in the etiology of several human diseases, including cancer, obesity, diabetes and cardiovascular and neurodegenerative diseases [113]. However, until now only one study investigated mitoepigenetic modifications in the peripheral blood of patients with MCI [71], and results suggest that this field of research deserves to be further investigated. Another issue that should be addressed in future studies is the investigation of environmental factors to which individuals have been exposed during their life, since they can play an important role in the etiology of AD [114]. In this context, epigenetic mechanisms, that are able to mediate the interaction between the genome and the environment, could provide a mechanistic explanation that might help our understanding of AD pathogenesis. Indeed, it is well-established that adverse environmental factors effects could be induced through the modulation of epigenetic mechanisms, and some authors believe that the epigenetic insult detected in AD patients has occurred in early life, during neurogenesis and synaptic formation, or that may be the consequence of life-long dietary habits, lifestyles, as well as occupational and environmental exposures that lead to age-related epigenetic drifts linked to dementia [115]. Considering that epigenetic markers have great plasticity, and could be reversed through lifestyle interventions, identification of modifiable environmental risk factors for dementia together with epigenetic biomarkers in easy-to-collect tissue could provide suggestions for new therapeutic approaches for AD, which can have profound implications for the economic cost of public health and individuals’ suffering.

## Figures and Tables

**Table 1:** Summary of DNA-methylation studies for early detection of AD.

Experimental Model	Diagnosis	Methodology for DNA Methylation Analyses	DNA Methylation End Point Investigated	Observation *	Reference
Blood/ 18 presymptomatic dementia and 18 HC with T2D	Neurological examination	HumanMethylation450 BeadChip	DNA methylation at genome-wide level	Eight DMPs associated with various genes, including *RPL13*, *RPL8*, *PAX2*, *KCNG2*	[40]
Blood/ 96 MCI and 96 HC from Uygur and Han Chinese populations	Neurological examination	Pyrosequencing	*KLOTHO* gene promoter	Increased *KLOTHO* methylation in the Han MCI, but not in the Uygur individuals. Higher *KLOTHO* methylation in Han MCI patients than Uygur MCI patients	[43]
Blood/ 96 MCI and 96 HC from Uygur and Han Chinese populations	Neurological examination	Pyrosequencing	*BDNF* promoter	No difference between MCI and HC in *BDNF* methylation	[44]
Blood/ 53 AD, 17 VaD, 32 mixed dementia, 47 MCI and 32 HC	Neurological examination	Imprint Methylated DNA Quantification Kit MDQ1	Global DNA methylation	No differences in global DNA methylation among groups	[52]
Blood/ 84 MCI 78 HC with T2D and 80 HC without T2D	Neurological examination	Methylation-specific PCR	5′-flanking region *SORL1* gene	The methylation ratio of MCI patients was higher compared to HC with and without T2D	[47]
Blood/ 30 AD, 28 aMCI and 30 HC	Neurological examination	HumanMethylation450 BeadChip and pyrosequencing	DNA methylation at genome-wide level and 4 CpG sites in *NCAPH2*/*LMF2* promoter region	*NCAPH2*/*LMF2* methylation lower in the aMCI and AD compared to HC. In the AD group methylation higher than in the aMCI. Negative correlation between methylation levels and MMSE score	[48]
Blood/ 30 AD, 28 aMCI and 30 HC	Neurological examination	MS-HRM	*COASY* and *SPINT1* gene promoter regions	DNA methylation in the two regions was increased in AD and aMCI as compared to controls	[50]
Blood/ 105 AD, 13 MCI and 10 HC	Neurological examination	Quantitative methylation-specific-PCR	*HMOX1* gene promoter	Lower methylation of *HMOX1* promoter in AD patients compared to MCI and HC, but no between MCI and HC	[53]
Blood/ 96 MCI and 96 HC from Uygur and Han Chinese populations	Neurological examination	Pyrosequencing	*OPRK1* and *OPRM1* genes	*OPRK1* hypermethylated in Han MCI females. *OPRM1* CpG1 hypermethylation and CpG2-4 hypomethylation associated with MCI risk in Uygur and Han, respectively	[45]
Blood/ 506 aMCI and 728 HC. After 5-year follow-up 128 aMCI converted to AD	Neurological examination	Pyrosequencing	Three CpG sites in the I promoter of *BDNF* gene and four CpG sites in the IV promoter of *BDNF* gene	Hypermethylation of two CpG sites in *BDNF* I promoter and of two CpG sites in *BDNF* IV promoter in MCI and in the conversion group. *BDNF* methylation higher in the aMCI with AA than that with AG or GG rs6265 genotype	[54,55]
Blood/ 24 MCI and 24 HC	Neurological examination	HumanMethylation450 BeadChip	DNA methylation at genome-wide level	Identified a number of non-significant DMPs associated with cognitive decline (most significant DMP resided in *BNC1*). Eight DMRs annotated to the HLA-DPA1/HLA-*DPB1*, *DRC1*, *PRKAA2*, *CALCB*, *CDH2*, *RTBDN*, *ZNF256* and *SHANK2* genes associated with cognitive decline	[58]
Blood/ 26 AD, 17 MCI and 24 HC	Neurological examination, CSF biomarkers	HumanMethylation450 BeadChip	*TOMM40*-*APOE*-*APOC2* locus	Differences in methylation levels observed between MCI and AD compared to controls. Methylation levels associated with CSF Aβ levels	[64]
Blood/ 102 MCI and 68 HC	Neurological examination	HumanMethylation450 BeadChip (in twelve subjects) and Sequenom EpiTyper	DNA methylation at genome-wide level and 25 CpG sites of the *NUDT15* gene and 17 CpG sites of the *TXNRD1* gene	*NUDT15* and *TXNRD1* hypermethylated in MCI. Several correlations between methylation and serum levels of folate, homocysteine, and oxidative biomarkers were observed	[62]
Blood/ 54 individuals that converted to AD (~4.5 years) and 42 HC	Neurological examination	HumanMethylation450 BeadChip	DNA methylation at genome-wide level	3 DMPs at baseline and 266 at follow-up, 15 and 21 DMRs associated with conversion to AD at baseline and to follow-up, respectively, 1 DMR, close to *GLIPR1L2*, hypermethylated at both the baseline and follow-up time points. Interestingly, a DMR close to the *OXT* gene detected also in the MTG	[63]
Blood/ 45 MCI and 45 HC	Neurological examination	Infinium^®^ MethylationEPIC BeadChip	DNA methylation at genome-wide level	Identified ten DMPs between controls and MCI annotated to *PKIB*, *KLHL29*, *SEPT9*, *OR2C3*, *CPLX3*, *BCL2L2-PABPN1*, and *CCNY* and four DMRs associated with *TMEM232*, *SLC17A8*, *ALOX12*, and *SEPT8*	[61]
Blood/ 43 MCI and 125 HC	Neurological examination	Methylation-specific PCR (qMSP)	*OGG1* and *DLST* genes	Methylation of *DLST* and *OGG1* genes not associated with MCI. DLST hypomethylation associated with MCI in the carriers of *APOE*ε4. Among the non-*APOE* ε4 carriers younger than 75, *OGG1* methylation levels associated with MCI	[46]
Blood/ 41 MCI and 59 HC	Neurological and neuroimaging examinations	Bisulfite Sequencing	*APOE* IV exon gene	Five CpG sites methylation levels were higher, while one CpG site was lower in MCI patients compared to control subjects	[65]
Blood at baseline and buccal samples at follow-up/ After 14-year follow-up period, 70 AD and 679 HC	Neurological examination	Sequenom EpiTyper	*BDNF* exon 1 promoter	Weak evidence of an association between blood methylation and dementia observed at one of 11 CpG sites investigated. Buccal methylation at two other sites associated with 14-year incident dementia cases prior to adjustment for multiple comparisons only with small effect size	[56]
Blood/ 73 individuals prior to dementia diagnosis and 87 HC; at 3 years follow-up 25 dementia cases and 24 HC	Neurological examination	MethylationEPIC BeadChip Array	DNA methylation at genome-wide level	Cases and controls differed in DNA methylome at the time of diagnosis, and pre-diagnosis, with a CpG associated with *GTF2A1* after correction for multiple testing	[57]
Blood/ 73 pre-AD and 87 HC. 25 AD and 24 HC after 3 years follow-up	Neurological examination	MethylationEPIC BeadChip Array	*APOE*, *APP*, *BDNF*, *PIN1*, *SNCA* and *TOMM40*	*APOE* and *TOMM40* methylation differed between pre-AD and HC. Several DMPs identified between AD and HC; greatest effect size detected in APP	[21]
Blood/ 151 AD, 22 aMCI, 21 VaD and 200 HC	Neurological examination	MS-HRM	*COASY* gene promoter	*COASY* hypermethylation in aMCI and AD	[51]
Blood/ 86 AD, 109 MCI and 89 HC; 38 MCI progressed to AD within 1 year	Neurological and neuroimaging examinations	HumanMethylation450 BeadChip and pyrosequencing	DNA methylation at genome-wide level and five CpG sites within *HOXB6* region	Nine DMRs associated with MCI-AD conversion. DMRs showing decreased methylation associated with *CPT1B* and *CHKB*, *TMEM184 A*, *KCNAB3*, *GABBR1*, *PRDM1*, *FLJ37453* and *OR56A3* and *TRIM5* genes. DMRs showing increased methylation associated with *SMC1B* and *RIBC2*, and *FIGN*	[23]
Blood/ 94 AD, 336 MCI and 223 HC	Neurological and neuroimaging examinations, CSF biomarkers	MethylationEPIC BeadChip Array	DNA methylation at genome-wide level	260, 91, and 137 DMPs, identified when comparing AD vs. HC, AD vs. MCI, and MCI vs. HC, respectively. The DMP that had the strongest association with MCI vs. HC was annotated to *CLIP4*, while the DMP that had the strongest association with MCI vs. AD was annotated to *NUCB2*	[66]
Blood/ 87 AD, 175 MCI and 162 HC	Neurological and neuroimaging examinations, CSF biomarkers	MethylationEPIC BeadChip Array	DNA methylation at genome-wide level	*PM20D1* hypomethylation in MCI, even more prominent in patients with mild to moderate AD. After 4 years *PM20D1* hypomethylation during MCI and early-stage AD reversed to hypermethylation in late-stage AD	[22]
Blood/ 330 SCD and 484 HC	Neurological examinations, CSF biomarkers	MethylTarget Sequencing	*BIN1* gene	*BIN1* hypomethylation in SCD. Hypomethylation of *BIN1* promoter associated with decreased CSF Aβ42, as well as increased p-tau/Aβ42 and t-tau/Aβ42 in total population, and with increased CSF p-tau and t-tau in the SCD subgroup	[69]
Blood/ 202 HC of which 56 converted to MCI; 317 MCI group of which 115 converted to AD	Neurological and neuroimaging examinations, CSF biomarkers	Infinium^®^ MethylationEPIC BeadChip	DNA methylation at genome-wide level	A DMP annotated to *RP11*-661A12.5 associated with the slope of cognitive decline from MCI to AD. Five DMRs related to the slope of cognitive decline from MCI to AD; the most significant DMR annotated to the gene *PM20D1*	[67]
Blood/ 34 HC of which 17 developed dementia within 4 years	Neurological examination	Infinium^®^ MethylationEPIC BeadChip	DNA methylation at genome-wide level	Several DMPs associated with various genes, including *PON1*, *AP2A2*, *MAGI2*, *POT1*, *ITGAX*, *PACSIN1*, *SLC2A8*, and *EIF4E*, as well as *HOXB6* and *PM20D1* associated with dementia development	[68]
Blood/ 18 early-stage AD, 70 advanced stage AD, 14 MCI and 105 HC	Neurological and neuroimaging examinations, CSF biomarkers	MS-HRM	Mitochondrial D-loop region	Higher D-loop methylation levels in MCI compared to HC and AD patients, as well as in HC compared to AD in advanced stages. Negative correlation between D-loop methylation levels and CSF p-tau	[71]

* All observations are statistically significant unless otherwise stated. Abbreviations: AD, Alzheimer’s disease; ADNI, Alzheimer’s Disease Neuroimaging Initiative; CSF, cerebrospinal fluid; DLST, dihydrolipoamide S-succinyltransferase; DMPs, differentially methylated positions; DMRs, differentially methylated regions; DSM-IV, the fourth edition of the Diagnostic and Statistical Manual of Mental Disorders; FAB, frontal assessment battery; HC, healthy controls; MCI, mild cognitive impairment; MMSE, mini-mental state examination; MoCA, Montreal Cognitive Assessment; MRI, magnetic resonance imaging; MS-HRM, Methylation-sensitive high-resolution melting; MTG, middle temporal gyrus; NINCDS-ADRDA, National Institute of Neurological and Communicative Disorders and Stroke and the Alzheimer’s Disease and Related Disorders Association criteria; OGG1, 8-oxoguanine DNA glycosylase 1; SCD, subjective cognitive decline; T2D, type 2 diabetes; VaD, vascular dementia.

**Table 2:** Summary of ncRNAs studies for early detection of AD.

Sample Type/ Study Cohort	Diagnosis	Methodology for ncRNAs Analyses	Observation *	Reference
Serum/ 75 MCI and 52 HC	Neurological examinations	RT-qPCR	Combination of hsa-let-7g-5p, hsa-miR-107 and hsa-miR-186-3p with diet and gut microbiota composition distinguished MCI from HC	[80]
Serum/ 127 AD, 30 MCI and 123 HC	Neurological examinations	Solexa sequencing and RT-qPCR	Low levels of miR-143 combined with high concentrations of miR-93 and miR-146a found in MCI subjects compared with HC	[81]
Plasma/ 50 MCI and 50 HC	Neurological examinations	RT-qPCR	Two sets of the miR-132 and miR-134 families differentiated MCI from HC with high specificity and sensitivity	[83]
Plasma/ 23 MCI and 30 HC	Neurological examinations	RT-qPCR	Two sets of miRNAs (hsa-miR-191 and hsa-miR-101, and hsa-miR-103 and hsa-miR-222) had high accuracy for MCI detection	[84]
Plasma/ 20 AD, 15 MCI and 15 HC	Neurological examinations and CSF biomarkers	RT-qPCR	Profile of six miRNAs detected AD at the early stage from HC	[85]
Serum/ 71 aMCI and 69 HC	Neurological examinations	RT-qPCR	Circulating miR-34c in patients with aMCI compared with HC, showing a positive correlation with MMSE	[86]
Plasma/ 97 AD, 116 aMCI and 81 HC	Neurological examinations	RT-qPCR	MiR-107 differentiated aMCI patients from HC with high sensitivity and specificity	[87]
Plasma/ 36 AD, 36 PAD and 36 HC	Neurological examinations and CSF biomarkers	RT-qPCR	MiR-43a-5p and miR-545-3p discriminated PAD from AD and HC	[88]
Plasma/ 65 aMCI and 55 HC	Neurological and neuroimaging examinations	Microarray sequencing	MiR-1185-2-3p, miR-1909-3p, miR-22-5p, miR-134-3p, and miR-107 discriminated aMCI from HC with high accuracy	[89]
Plasma/ 45 AD and 36 HC	Neurological examinations	RT-qPCR	BACE1-AS discriminated full-AD, pre-AD and HC subgroups	[95]
Plasma/ 70 AD and 90 HC	Neurological examinations	RT-qPCR	High levels of 51A found in AD compared with HC, showing a negative correlation with MMSE	[100]
Plasma/ 50 AD and 50 HC	Neurological examinations	RT-qPCR	Levels of NEAT1 differentiated MCI and advanced-AD from HC whereas levels of BC200 discriminated pre-clinical AD from HC	[101]

* All observations are statistically significant. Abbreviations: AD, Alzheimer’s disease; aMCI, amnestic mild cognitive impairment; HC, healthy controls; MCI, mild cognitive impairment; MMSE, mini-mental state examination; PAD, preclinical Alzheimer’s disease; RT-qPCR, quantitative reverse transcription real-time PCR.

## Data Availability

Not applicable.
